# The Role of Serum Level of Interleukin-6 in Severity of Pulmonary Complications of Sulfur Mustard Injuries

**Published:** 2014-07

**Authors:** Majid Shohrati, Ali Amini-Harandi, Bita Najafian, Amin Saburi, Mostafa Ghanei

**Affiliations:** 1 Chemical Injuries Research Center, Baqiyatallah University of Medical Sciences, Tehran, Iran;; 2 Department of Pediatrics, Faculty of Medicine, Baqiyatallah University of Medical Sciences, Tehran, Iran;; 3 Health Research Center, Baqiyatallah University of Medical Sciences, Tehran, Iran

**Keywords:** Bronchiolitis, Mustard gas, Interleukin-6, Cytokines

## Abstract

Diverse studies suggest that interleukin-6 (IL6), as a member of cytokines family, has a major role in inflammatory processes of airways and lungs. In this study, an attempt was made to determine the serum level of IL6 in sulfur mustard (SM) injured patients and its comparison with controls. The measured IL6 mean level in patients with chemical injuries (0.76±0.3 ng/ml) was significantly higher than the control group’s mean level (0.34±0.12 ng/ml). Furthermore, patients with moderate to severe symptoms had a serum level of (0.95±0.92 ng/ml) which was significantly higher than mild (0.47±0.54) and control (0.34±0.12) groups.

The outcome of this research program demonstrates that an increase in serum level of IL6 can have a role in pulmonary complications of SM, similar to other well defined pulmonary diseases.  However, further studies are required to clarify the role and mechanism of IL6 in such patients.

## Introduction

The role of cytokines has become more important in the immune and inflammatory processes. One of those important cytokines is interleukin-6 (IL6), which is produced by mononuclear phagocyte, fibroblasts, endothelial cells and few other cells in response to microbes, foreign and inflammatory agents. The active form of IL6 is a homodimer where IL6 does its function by a receptor that consists of a cytokines-binding protein and a signal-transducing compound.^1^ This compound activates a JAK/STAT signaling pathway as well as the signaling pathway of other cytokine receptors. IL6 has various activities among which stimulating synthesis of acute-phase protein by liver cells is one of its main roles that contributes to the systemic effects of inflammation. Additionally, IL6 stimulates production of neutrophils, as important inflammatory cells in acute phase, from bone marrow and it also stimulates the growth of B lymphocytes in immune responses.^1^

One of the specific functions of IL6_,_ which has recently been discovered, is its prime role in the inflammatory processes of lung disease, particularly chronic obstructive pulmonary diseases (COPD), bronchiolitis and asthma. It is reported that the change in the serum level of IL6 can affect exacerbations of COPD patients.^2^ Similar changes in IL6 level has been observed in their sputum and condensate exhalation.^3^^,^^4^ Close correlation between IL6 serum level and main symptoms of COPD, such as severe dyspnea and pulmonary dysfunction, has been reported.^5^^,^^6^ Besides, cigarette smoke which is an important inflammatory agent, contribute its inflammatory effect by stimulating IL6 and few other cytokines production from bronchial epithelial cells.^7^^,^^8^ Furthermore, in asthma where complex inflammatory pathways lead to diverse manifestations, serum level of IL6, its soluble receptor and its gene-expression are significantly correlated with asthma attacks.^9^^-^^12^ Chemical particles in polluted air create similar inflammatory process in human airways and are closely correlated with mRNA and serum level of IL6.^13^ The majority of the Iran-Iraq war victims subjected to sulfur mustard (SM) chemical gas attacks, suffer from chronic pulmonary complications^14^ where pulmonary complication is one of the most important impediments of chemical gases specially SM.^15^^-^^17^

Some of the main pulmonary complications of SM are namely; COPD, bronchitis, emphysema, asthma, bronchectasia and more commonly Bronchiolitis Obliterans (BO).^18^ Unfortunately, a majority of the regular therapies for war victims subjected to SM chemical gas attack has been unsatisfactory.^19^^,^^20^ Consequently, the main focus of the current research program was to reveal new mechanisms and to offer effective therapies in pulmonary lesions of SM. This is done by investigating the relationship between IL6 serum level and chronic pulmonary complications of SM like COPD in such patients.

## Materials and Methods

A cross sectional study on the chemical victims of SM with pulmonary symptoms was carried out once a written agreement from the patients at the Baqiyatallah hospital, Tehran, Iran was obtained. Patients with documented exposure to SM during the Iran-Iraq conflict in 1980’s who displayed symptoms of SM lung injuries (including productive cough, dyspnea in exertion, chest tightness, etc) with diagnosis of BO, were recruited. The patients were divided into two groups each consisting of thirty individuals. The first group included thirty patients with mild to moderate pulmonary symptoms and the second group of thirty patients with moderate to severe pulmonary symptoms. Recent pulmonary and airways infections, as well as other toxicants such as detergent exposure, were used as the exclusion criteria. Next to the above sixty patients, a third group of thirty individuals without any history of lung diseases but with matched age and gender was formed as a reference for comparisons. Categorization of war veteran patients into “mild to moderate” and “moderate to severe” was according to the severity of their pulmonary symptoms as well as their spirometric results based on the American Thoracic Society (ATS) guideline.^21^ The sample size was calculated according to the formula:

$$n=\frac{2{\left({\text{Z}}_{\frac{\alpha }{2}}+{\text{Z}}_{\beta }\right)}^{2}}{{\text{d}}^{2}}$$

The inclusion criteria were; 1) Pulmonary exposure with SM, 2) Diagnosis of BO with physical exam, spirometry, and high resolute computed tomography 3) Age range between 20-70 and 4) Patients approval. The exclusion criteria were; 1) Any malignancy, 2) Pneumonia or bacterial bronchitis in the recent month, 3) Addiction to cigarette or other opioids and 4) Any inflammatory disorders such as rheumatologic diseases or etc.

Following a routine physical examination and a pulmonary function test (PFT), patients’ blood samples were taken with a heparinated syringe and then centrifuged. The purified plasma was kept in a 1 milliliter (mL) tube in -70°C until the start of the actual measurement. Then, step by step, the IL6 kit (Cayman Chemical Company, Michigan, USA) stages were followed, from which results were taken with a ELISA reader in the wave of 450nm (with the reference of 620 nanometer). The complete set of data was analyzed with SPSS software with ANOVA, and *t* test.

## Results

**Figure 1:**
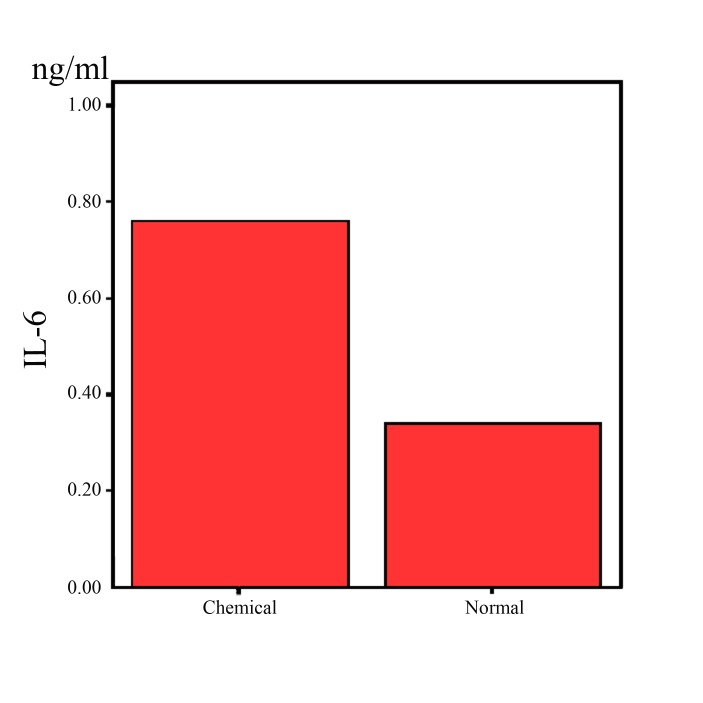
Serum level of IL6 in cases and controls.

As mentioned above, ninety individuals in three groups were studied. Thirty patients with “mild to moderate” pulmonary symptoms, an additional thirty patients with “moderate to severe” pulmonary symptoms and thirty individuals as control group were categorized. The measured IL6 mean concentration in patients with chemical exposure was 0.76±0.3 picogram (pg)/ml, being significantly higher than those in the control group 0.34±0.12 (P=0.17) as shown in figure 1.

**Figure 2:**
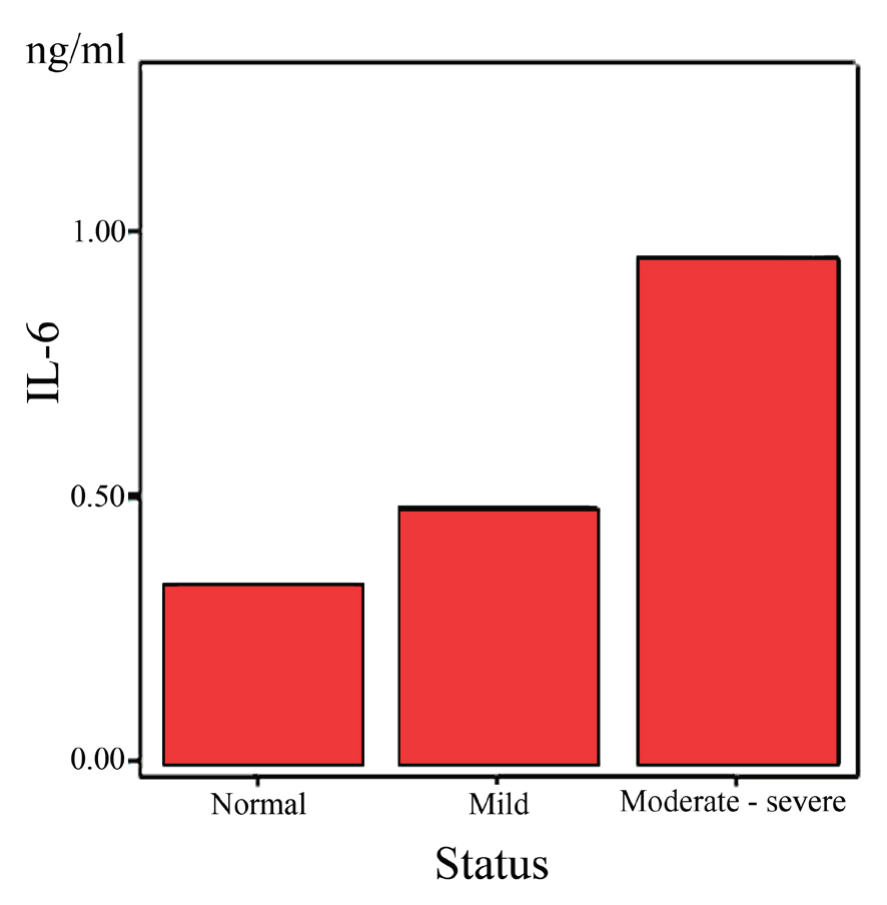
Serum level of IL6 in terms of severity of disease.

In figure 2, analysis of data from patients with moderate to severe pulmonary symptoms revealed that the IL6 mean concentration in this group (0.95±0.92 pg/ml) was significantly more than those patients with mild to moderate symptoms (0.47±0.54 pg/ml) as well as those in the control group (0.34±0.12 pg/ml, P=0.004).

## Discussion

In this study, the measured IL6 mean level in patients with chemical exposure was significantly higher than mean level of the control group. Moreover, patients with moderate to severe symptoms had significantly higher level of serum IL6 compared with the mild or control groups. Few studies have shown that the serum level of IL6 increases in exacerbation periods of patients with COPD. This causes the elevation in the serum fibrinogen level and cardiovascular problems in such patients.^2^

Survey of condensed exhaled air with an enzyme immunoassay kit in COPD patients, has revealed high level of IL6 in those patients in comparison with normal non-smokers.^3^ Another study on the induced sputum of COPD patients suggests that elevated levels of IL6, IL8 and tumor necrosing factor alpha (TNFa) in such patients are correlated with pathogenesis and progression of COPD.^4^ In other research, raised IL6 level is connected with fatigue of healthy people, especially athletes.^5^ This result can be extended to severe dyspnea and exhaustion of COPD patients. Other research has shown significant level increase of IL6 and TNFa transcripts (with real-time PCR and ELISA) in COPD patients in comparison with the control group.^6^

The main advantage of the current research program is the study on humans rather than animal. As noted in the reference list,^22^^-^^24^ there are a number of articles regarding serum and BAL fluid levels of some cytokines like IL6 (with ELISA) after exposure to SM. However, these are restricted to studies on animal cases. An investigation on animal cases has shown enhanced level of IL6 in the serum and BAL fluid of rats after exposure to SM.^22^ Clearly in this study the most elevation is for IL_9_. This interleukin (IL_9_) can be the focus for future studies on human patients with chemical exposure. Inflammation, particularly chronic inflammation, is one of the main pathogenesis of SM induced lung disorders. After acute exposure to SM, the repair process in the lungs and its airways as the main target of toxicants is activated but it would not control the inflammation.^25^ On the other hand, chronic inflammation decreases the ability of epithelium and pulmonary tissue to properly reconstruct. Low supply of anti-oxidant could induce such imbalance between inflammation and tissue repair. Therefore, the inflammation as well as the inflammatory molecules such as IL6 is the result of imbalance between the ineffective cell repair and the anti-oxidant insufficiency.^26^

In other studies, the inflammatory effects of cigarette smoking and the main role of IL6 in inducing bronchial inflammation has been presented. The inhibitory effect of NAC (N-acetyl-L-cysteine) on releasing IL6 from bronchial epithelial cells has been shown^7^ which in turn could pave the way for a new treatment approach for chemically exposed patients with COPD manifestations.

In a similar animal study the increased levels of IL6, IL8 and LPO (lipoperoxide) in smokers with chronic bronchitis is compared with non-smokers. The study also shows the protective effects of B-carotene intake in reducing serum and tissue levels of IL6, IL8 and LPO and in alleviation of pathologic changes in chronic bronchitis.^8^ This could be helpful in treatment plans for chemically exposed patients.

Genetic study on the effect of chemical particles in polluted air shows enhanced IL6-gene expression in the pulmonary epithelial cells which are exposed to such particles.^13^ Furthermore, significant elevation of serum level of IL6 and its soluble receptor during asthmatic attacks is reported in a few studies.^9^^,^^10^^,^^12^

The literature review presented in table 1, highlights similar studies to the current research program. Results presented by Attaran et al. concur with the data from this study but Pourfarzam et al.’s finding is different.^23^^,^^24^ Pourfarzam demonstrated that serum level of IL6 is lower in SM injured patients than controls and that the IL6 is associated with wheezing. However, Attaran showed that IL6 level is higher in SM injured patients. Attaran also declares significant correlation between IL6 level and global initiative for chronic obstructive lung disease stage, and BODE index (including body mass index, obstruction, dyspnea, and exercise capacity) and FEV1.^23^^,^^24^ It seems that the difference between our findings and those of Pourfarzam are due to the status of the patients (stable or in exacerbation), severity of diseases and the type of diseases (COPD vs. BO).^24^

**Table 1:** Iranian reports about Serum Level of IL6 in SM injured patients with chronic lung sequels

**Study**	**year**	**case vs. control**	**Correlation**	**Other findings**
Pourfarzam et al.^24^	2009	348 vs. 120	Significantly lower than controls	IL6 was associated with wheezing
Attaran et al.^23^	2010	50 vs. 30	Significantly higher than controls	Significant correlation between IL6 level and Global Initiative for Chronic Obstructive Lung Disease stage, and BODE index (including body mass index, obstruction, dyspnea, and exercise capacity) and FEV1
Present	2012	60 vs. 30	Significantly higher than controls	Serum IL6 level was higher in severe cases vs. moderate or mild cases.

Vs.: Versus; IL6: Interleukin 6; BODE index: body mass index, obstruction, dyspnea, and exercise capacity; FEV1: Forced Expiratory Volume in 1^st^ second

Another interesting research^11^ has demonstrated that there is a balance between IL6 pro and anti-inflammatory actions during airways inflammation in such a way that an increased level of soluble form of IL6-receptor leads to raise airway inflammation. On the other hand, blockade of membrane form of IL6-receptor leads to increase regulatory T cells and prevents overactive inflammatory responses in airways. This finding could be useful in defining a treatment plan.

On a final note, prior to selecting chemically exposed patients and the matched controls no ambiguous factors that could affect present findings were anticipated. However, due to probable unforeseen uncertainties, multivariate analysis is not preformed. This could be considered as a limitation of this study. 

## Conclusion

This research program shows that the mean serum level of IL6 (with ELISA) in patients exposed to mustard chemical with COPD manifestations is significantly higher than those in the control group. It is also shown that the proportion of this elevation is distinctly related with the degree of the disease and being clearly higher in patients with moderate to severe symptoms than those with mild to moderate symptoms. Simulation of the latter genomic study could pave the way in finding new inflammatory pathways of other chemical gases similar to mustard. 
